# Checkpoint Inhibitors Immunotherapy in Metastatic Melanoma: When to Stop Treatment?

**DOI:** 10.3390/biomedicines10102424

**Published:** 2022-09-28

**Authors:** Ivana De Risi, Angela Monica Sciacovelli, Michele Guida

**Affiliations:** Rare Tumors and Melanoma Unit, IRCCS Istituto dei Tumori “Giovanni Paolo II”, 70124 Bari, Italy

**Keywords:** metastatic melanoma, immune checkpoint inhibitors, therapy discontinuation

## Abstract

Background: Immune checkpoint inhibition (ICI) has significantly improved the survival of metastatic melanoma (MM) with a significant proportion of patients obtaining long-lasting responses. However, ICI also exposes patients to new, heavy, and sometimes irreversible toxicities. Thus, identifying the minimal amount of treatment time is extremely urgent. Methods: We researched English peer-reviewed literature from electronic databases (MEDLINE and PubMed) until July 2022 with the aim of evaluating the clinical outcomes after the cessation of ICI therapy due to elective study plans, clinician–patient sharing, and adverse events. Results: Although most of the data are from retrospective studies, considering that most patients with major responses maintain it after treatment cessation, it is proposed that for complete response (CR)/near CR, a further six months of therapy after best response may be considered enough. For partial response (PR) or stable disease (SD), treatment must be continued for at least 2 years and, in some cases, indefinitely, based on residual disease, the patient’s will, and the toxic profile. Of note, in spite of the best response, 25–30% of patients relapsed, and, when retreated, responded far less than in front-line treatment. Conclusions: Most of the data being from retrospective and heterogeneous experiences, their grade of evidence is limited and no consensus has been reached on the optimal treatment duration. Controlled prospective studies are needed.

## 1. Introduction

Targeted therapy with BRAF/MEK inhibitors and immune checkpoint inhibition (ICI) provides long-term overall survival (OS) benefits in patients with metastatic melanoma (MM). The median OS of various available anti-BRAF/anti-MEK combinations is 23–34 months and that of anti-PD-1 (programmed death 1 receptor) single agent is 33–37 months with a 5-year OS rate of about 35% for targeted therapy and about 45%, for anti-PD-1 immunotherapy [1,2,3]. Combined ICI nivolumab plus the anti-cytotoxic T lymphocyte antigen-4 (CTLA-4) ipilimumab yielded an even superior clinical benefit compared to each agent used alone with a median OS of about 72 months and a 5-year OS rate of 52% [4].

Despite the approval for long-term use, the optimal treatment duration providing the maximum anti-tumor effect and minimizing the duration of treatment is still a matter of debate [5]. The FDA label for nivolumab and pembrolizumab allows for indefinite therapy, which, for many patients represents overtreatment. In clinical trials, anti-PD-1 antibodies are typically administered continuously over 2 years or until reaching a progression of the disease or unacceptable toxicity. In daily practice, treatment is often discontinued due to treatment-limiting toxicity (TLT) or electively, several months to years following a major tumor response [6,7].

Understanding the long-term outcomes after treatment discontinuation is of great importance for patient and physician decisions. At present, data on responding patients who discontinue therapy are scarce and widely variable [8,9,10,11]. Real-life evidence for durable responses after treatment cessation has consistently accumulated in recent years and this questions the need for prolonged treatment in responding patients [5,11,12].

Another important aspect concerns the number of patients who relapse and undergo a new anti-PD-1 treatment. To date, data on retreatment are lacking and based only on small cohorts of patients [8,9,13,14]. 

The purpose of this review was to analyze clinical experiences carried out both from controlled studies and real-life experiences of the discontinuation of ICI therapy in the absence of disease progression with the aim of assessing the characteristics of long-term benefits after stopping therapy and to help clinicians to consider the optimal duration of treatment in a specific patient and therapeutic context. Data regarding retreatment with anti-PD-1 at recurrence have been also reported.

## 2. Materials and Methods

We researched English peer-reviewed literature (clinical trials, real-life retrospective studies, scientific reviews, and reference lists of respective articles) from electronic databases (MEDLINE and PubMed, until 31 July 2022). 

We searched both controlled clinical trials and real-life studies, using immunotherapy, treatment discontinuation, checkpoint inhibitor toxicity, and metastatic melanoma as keywords. We excluded all studies that involved the discontinuation of treatment with anti-CTLA-4, or other therapies other than anti-PD-1, and the interruption of treatment due to disease progression We described patient outcomes in terms of relapse rate, probability of disease-free survival (DFS) at 12 and 24 months (when present), and response rate to retreatment. A flow chart of the systematic literature search was made according to PRISMA guidelines (Supplementary Figure S1). The registration code is 338888 of 11 June 2022.

## 3. Results

### 3.1. Long-Term Benefit with Checkpoint Inhibitor Immunotherapy

In the Keynote 006 open-label randomized phase-3 study including 834 patients and comparing pembrolizumab vs. ipilimumab, in patients with stable disease (SD) or better who completed the entire plan of 2 years of pembrolizumab, the 5-year PFS rate and OS rate are 70% and 93%, respectively [1]. A recent update of this study at 7 years of follow-up has confirmed the long-term OS benefit of pembrolizumab with an OS rate of 37.8%, regardless of BRAF status, prior BRAF inhibitor therapy, or poor prognostic characteristics [4].

Also, the updated follow-up at 6.5-year of the CheckMate 067 study, comparing nivolumab alone or combined with ipilimumab vs. ipilimumab, reported a median OS of 72 months for nivolumab plus ipilimumab, 36.9 for nivolumab, and 19.9 for ipilimumab as a single agent. The OS rate at that time was 49%, 42%, and 23%, respectively [15].

Of note, the long-term benefit for patients treated both with targeted therapy or checkpoint inhibitors was closely correlated with the quality of response. In BRAF-mutated patients treated with dabrafenib and trametinib, the 5-year OS according to the best response was 71%, 32%, and 16% for complete response (CR), partial response (PR) and SD, respectively [16]. A similar trend was reported in the Keynote 006 study with a 7-year OS rate of 85.2% for CR, 61.8% for PR, and 25.9% for SD [4]. Finally, in the CheckMate 066, 067, and 069 pooled analysis, the 5-year OS rate for the single-agent nivolumab group was 86% for patients who reached a CR and 54% for patients who did not reach a CR, whereas in the nivolumab plus ipilimumab group it was 85% and 68%, respectively. Interestingly, patients who achieved CR had similar OS behavior regardless of the treatment group [17].

Regarding ICI therapy, it is also reported that patients who discontinued treatment for adverse events (AEs), which is about 10% of patients treated with anti-PD-1 single agent [1], and near 40% of those treated with anti-PD-1 plus anti-CTLA-4 [2], maintain clinical benefits similar to those who continued the treatment with ICI. A comprehensive pooled landmark analysis of data from the Keynote-001, Keynote-002, and Keynote-006 studies reporting the long-term (3.5 years) safety profile of pembrolizumab demonstrated that treatment-related AEs were generally mild to moderate and that the efficacy of pembrolizumab was similar in the 79 patients who experienced AEs with respect to the 384 patients who did not. Moreover, the efficacy of pembrolizumab was also similar in patients who received or did not receive systemic corticosteroids to manage immune AEs [18]. Similar data were obtained with nivolumab plus ipilimumab: the pooled analysis of CheckMate 069 and 067 at a median length of follow-up of 21.3 months reported a median PFS of 16.7 months for 176 patients who discontinued therapy due to an AE vs. 10.8 for the 233 patients without AEs [19]. The possible explanation is that the mechanisms underlying the AEs are also responsible for a more potent antitumor immune response that persists even after treatment is stopped. Nevertheless, real-life experiences have reported a poorer survival in patients who discontinued due to AEs compared with those who electively discontinued anti-PD-1 therapy, as reported below.

### 3.2. Experiences of Discontinuation in the Absence of Disease Progression

We analyzed 10 studies focused on the discontinuation of anti-PD-1 therapy, published from 2018 to 2022, involving a total of 1199 patients. Two prospective clinical trials [1,9,20] and eight retrospective studies [21,22,23,24,25,26,27,28] were evaluated. Responses to checkpoint inhibitors were mainly assessed according to RECIST 1.1 criteria [1,9,20,21,24,25,27,28]. Only in one study were immuno-related response criteria used [22]. Exceptions are two studies, one in which response assessment was not strictly related to RECIST 1.1 criteria but to physician judgment [23], and one in which the method of response assessment was not specified [26]. We also evaluated the percentage of relapsed patients and the response to re-treatment with anti-PD-1. To this aim, we analyzed nine studies, including one prospective clinical trial [1] and eight retrospective studies [22,23,24,25,26,27,29,30].

In the phase-1 Keynote-001 study using the anti-PD-1 pembrolizumab in 655 patients, an amendment to the protocol allowed treatment discontinuation for patients with CR if they had at least 6 months of treatment, a CR confirmed on two consecutive CT scans, and two infusions of therapy after confirmation of response [9,20]. At 5 years of follow-up, 105 patients obtained a CR, of which 14 continued treatment and 91 stopped therapy (67 patients due to clinician–patient joint decision, 12 patients due to AEs, 7 patients due to clinical decision, 3 patients due to their own choice, and 2 patients due to subsequent progression). For the 67 patients who suspended treatment due to a joint decision, the median time of treatment was 23 months (range of 8 to 44 months). Of these, two (3%) patients died for reasons unrelated to study treatment or progression, four (6%) patients progressed, and sixty-one (91%) patients maintained the CR after a median time of therapy discontinuation of 22 months. For the entire group of responders, the estimated 24-month DFS was 90.9%, whereas for the 91 patients who discontinued therapy it was 85.8% [9,20].

The Keynote-006 study, an open-label, randomized, phase-3 study comparing pembrolizumab at two different schedules vs. ipilimumab, pembrolizumab treatment, could be continued for up to 24 months. Patients with a confirmed CR who received pembrolizumab for at least 6 months could discontinue therapy if they received two or more doses beyond the CR. Patients who discontinued pembrolizumab with SD or better after receiving at least 24 months of pembrolizumab or discontinued with CR after at least 6 months of pembrolizumab and then progressed could receive a second course of an additional 17 cycles of pembrolizumab [1]. Of the 556 patients treated with pembrolizumab, 103 completed the 2 years of planned treatment, and of these, 21 (20.4%) patients had a CR, 69 (67%) a PR, and 13 (12.6%) an SD. After a median follow-up from the therapy discontinuation of 34.2 months, 76% of patients with CR, 77% of patients with PR, and 54% of patients with SD maintained their response after the end of therapy. Moreover, patients with SD progressed earlier than those with CR or PR. Of note, eight patients who had PR before therapy discontinuation became complete responders after discontinuation. The estimated 24-month DFS was 78.4% for all 103 patients, 85.4% for patients with CR, 82.3% for patients with PR, and 39.9% for patients with SD. Nevertheless, 23 patients with CR who completed at least 6 months of therapy but did not complete the 24 months of planned treatment had a 24-month DFS of 86.4%, similar to patients with CR who completed the 2 years of treatment [1].

Beyond these data from controlled studies, new evidence is accumulating from retrospective real-life experiences. Dimitriou et al. recently reported a European multicenter experience of 125 patients with advanced melanoma with and without brain metastases treated either with anti-PD-1 monotherapy (97 patients) or combined with anti-CTLA-4 (28 patients) [21]. Eighty-six patients electively discontinued the treatment after CR (first group), thirty-three patients due to TLT, and six due to the investigator’s decision (second group). For the first group, the median duration of treatment was 22 months (range 5–49), the median time to CR was 9 months (range 2–47), and the median treatment after CR was 8 months (range 0–40). For the group of patients who discontinued treatment due to toxicity or due to the investigator’s decision, the median treatment time was 3 months (range 0–36). Of the 86 patients who electively discontinued the treatment for CR, only 7 (8%) had a recurrence of the disease (two had been treated with combined anti-CTLA-4/anti-PD-1 and five with anti-PD-1 alone) and only 3 (7.7%) of the 39 patients who discontinued treatment due to toxicity or investigator’s choice (one previously treated with combined anti-CTLA-4/anti-PD-1 and two with anti-PD-1 alone). Median off-treatment response time (time between last immunotherapy dose to disease progression or last follow-up) was 19 months (range 0–42) and 25 months (range 0–66) for the first and second groups, respectively. It should be noted that in this study, not all patients were treated with anti-PD-1 alone and not all were treated as the first line. In fact, 32.3% of patients received one previous line, 19.2% two lines, and 1.6% up to four lines prior to immunotherapy. Interestingly, among the 25 patients with cerebral metastases, only 3 had a recurrence of disease (two intracranial treated with locoregional therapy) and none progressed at the cut-off of data. Moreover, in this study, at a median follow-up of 38 months, median PFS and OS were not reached and the 3-year OS was estimated at 90%. The authors concluded that treatment discontinuation is feasible in patients obtaining CR, including those with brain metastases, and that the efficacy outcomes seemed to be similar regardless of the reason for discontinuation [21].

In another retrospective, multicenter, real-world cohort study from 14 medical centers across Europe and Australia, Jansen et al., investigated the outcomes of 185 patients with advanced melanoma treated with anti-PD-1 monotherapy administered as first-line in 80 (43%) patients [22]. All patients interrupted treatment by the joint decision of the investigators and the patients in the absence of progression or TLT. The best overall response (BOR) included 117 CR (63%), 44 PR (24%), and 16 SD (9%), and 8 (4%) patients were non-evaluable. The median duration of treatment was lower than in other clinical trials (12 months, range 0.7–43). In detail, CR patients were treated for a median time of 11 months, PR patients for 15 months, and SD patients for 14 months. After a median follow-up of 18 months after treatment interruption, the relapse rate was 22% (40 patients) for the entire cohort, 14% (16 patients) for CR patients, 32% (14 patients) for PR patients, and 50% (8 patients) for SD. Multivariate analysis, including prognostic indicators (stage of the disease and previous therapies), did not detect any significant association between relapse and duration of the treatment or clinical characteristics. The median PFS was achieved only for patients with an SD of 16 months. When PFS was stratified according to the treatment duration (<6 vs. 6–9 months vs. 9–12 months vs. 12–18 months vs. >18 months), median PFS after discontinuation was not reached in any of the subgroups, and no significant difference was found between these groups. The authors found a statistically significant difference in PFS only for CR patients with a longer PFS for those treated >6 months vs. those treated <6 months (not reached vs. 18.9 months). No difference was found among patients treated for more than 6 months. The main message from these authors is that in patients obtaining a CR after treatment for >6 months, the risk of relapse after treatment discontinuation was very low [22].

In a more recent retrospective study, van Zeijl et al. reported an experience in the Netherlands of 324 patients homogeneously treated with anti-PD-1 as first-line therapy and who discontinued therapy in the absence of progressive disease [23]. At the time of the discontinuation of therapy, 90 (28%) patients had a CR, 190 (59%) a PR, and 44 (14%) an SD. For CR, patients the most common reasons for discontinuation were medical/patient joint decisions in 67 (70%) patients and AEs in 7 (7.8%) patients. In patients with PR, 98 (52%) of them discontinued by joint decision and 61 (32%) for AEs. In patients with SD, AEs were the most frequently reported reason for anti-PD-1 discontinuation (*n* = 21, 48%), followed by joint decision (*n* = 15, 34%). The median duration of treatment was 12 months for patients with CR, 13 months for patients with PR, and 15 months for SD. Eighty-seven (27%) patients had a recurrence of disease (16 patients with CR at the time of discontinuation, 52 with PR, and 19 with SD). In patients with CR, PR, and SD at the time of anti-PD-1 discontinuation, the median follow-up time after anti-PD-1 discontinuation was 18, 16, and 17 months. The median PFS was not reached for patients with CR and PR, whereas it was 10 months for patients with SD. The probability of PFS at 12 and 24 months after the discontinuation of anti-PD-1 for patients with CR was 86% and 64%, respectively; for patients with PR, it was 70% and 53%, respectively; and for patients with SD, it was 48% and 31%, respectively. Of note, the PFS of patients with CR was considerably lower than in controlled studies. This may be due to the fact that patients who discontinued treatment for AEs were also considered, in the presence of unfavorable patient and/or disease characteristics, and in the short period of only 1.1 months of therapy from BOR. Moreover, the authors found a better PFS for patients who had an elective interruption of treatment and PR compared to those who discontinued treatment for toxicity, probably due to the shorter treatment median time in the AEs group (6.9 months for CR, 7.2 months for PR, and 3.5 months for SD) than those who electively stopped treatment (12 months for CR, 13 months for PR, and 11 months for SD). Of note, in this study, PFS was not stratified based on the treatment time. Survival outcomes of patients with a PR and CR were similar when anti-PD-1 discontinuation was not due to adverse events [23]. In a retrospective monocentric study from the USA, Pokorny et al. analyzed data of 52 patients treated as the first-line with anti-PD-1 for 1 year (at least 6 months and not more than 18 months) and then stopped treatment after an investigator–patient joint decision [24]. In this cohort, patients who discontinued treatment due to PD or immune-related AEs were excluded. The median time of treatment was 11.1 months. At discontinuation, the BOR was CR in 13 (25%) patients, PR in 28 (53.8%) patients, and SD in 11 (21.2%) patients. The relapse rate to a median follow-up of 20.5 months from discontinuation was globally 25%, including 15.3% of patients who obtained a CR, 25% of those with PR, and 36.3% of those with SD. The median PFS for relapsed patients was 3.9 months (range 0.7–30.9). In contrast to Jansen [22], this study reported a significant correlation between specific disease characteristics and the likelihood of relapse in multivariate analysis. The authors found strong evidence that younger age (*p* = 0.037), history of brain metastasis (*p* = 0.009), and greater post-PD-1 lactate dehydrogenase (LDH) (*p* = 0.032) were associated with an earlier time of progression. The median PFS after treatment discontinuation was not reached. The authors concluded that, after 1 year of anti-PD-1 therapy, the majority of patients remained without progression after long-term follow-up, even patients with residual disease on imaging. They reported no correlation between treatment time and survival, probably because the treatment period was limited to 1 year. Unlike other studies, there is also a lack of estimation analysis of PFS and OS at 12 and 24 months [24].

In another monocentric retrospective study at Memorial Sloan Kettering Cancer Center of New York, Betof Warner et al. analyzed the clinical outcomes of 396 patients who interrupted therapy with anti-PD-1 or anti-PD-1/anti-CTLA-4 combination for all reasons, including disease progression (196 patients) and having at least 3 months of follow-up after discontinuation [25]. Among the entire cohort of patients, 102 had a CR as the best overall response (25.8%), with 18 patients considered as having CR by the clinician but having a residual tumor in the radiological analysis. In patients with CR, the median duration of treatment was 9.4 months, and the causes of treatment interruption were CR (72 patients), TLT (24 pts), PD (3 patients), completion of the clinical trial (1 patient), and other (2 patients). After a median follow-up of 21.1 months from CR, 23 patients (22.5%) had a recurrence of disease. The probability of being alive and not needing additional therapy for melanoma recurrence at 3 years was 72.1%. There was no significant association between treatment duration and relapse risk. In multivariable analysis, CR was associated with M1b disease and cutaneous vs. mucosal or acral primaries. Considering the 27% treatment failure at 3 years and the infrequent responses to retreatment, the authors concluded that the optimal duration of treatment after CR is yet to be established [25]. 

In another real-life, single-site experience from Israel, Asher et al. identified 106 patients with MM treated with immunotherapy (anti-PD-1 monotherapy in 81% of patients or in combination with ipilimumab in 19% of patients) for a median of 15.2 months (range, 0.7–42.3) and who discontinued treatment in the absence of disease progression [26]. Eighty (75.5%) patients received immunotherapy as the first line and 26 (24.5%) as the advanced treatment line (15 ipilimumab, 6 targeted therapy, 3 pembrolizumab, 2 ipilimumab and nivolumab)**.** Sixty patients discontinued treatment for toxicity, 32 for CR, and 14 for a long-term PR. Eighty patients (75.5%) had a CR as the BOR, 22 (20.7%) had a PR, and 4 (3.8%) had an SD. After a median follow-up from the interruption of therapy of 20.8 months, 34 (32%) patients had disease recurrence. The median time to progression was 8.5 months (range, 1.5–37). Nineteen (24%) patients with CR had a relapse of disease compared to 15 (57.7%) non-CR patients. Thus, the authors concluded that patients reaching CR had a significantly lower risk of disease progression than non-CR patients (OR 0.31; *p* = 0.02). They also found a higher likelihood of progression for patients who received previous treatments than patients treated in the front line (OR 2.8, *p* = 0.027). Specifically, patients with non-CR as the best response and patients treated in an advanced-line setting should be treated for longer periods, and elective discontinuation should not take place prior to 18 months. In a multivariate analysis, the authors found a correlation between PFS and best response (HR 2.46), line of treatment (HR 2.20), and treatment duration (HR = 0.98). The median PFS was not achieved for patients with CR, while for patients with PR it was 36.5 months, and 12.8 months for patients with SD. Moreover, the median OS was not reached for patients with CR or PR, whereas it was 24.6 months for patients with SD. Patients who had received previous treatment lines experienced a greater likelihood of progression after the anti-PD-1 interruption. PFS, however, was not significantly affected by the treatment line. The 72 non-progressed patients had a median duration of treatment statistically longer than patients who had disease progression (15.8 vs. 8.9 months). Calculating the hazard ratio at 3-month intervals, the authors found that the optimal duration of treatment was between 18 and 24 months [26], in contrast with Jensen et al., who found no significant differences between the duration of treatment at 12 or 18 months [22].

In a recent retrospective study from France, Valentin et al. selected 65 patients with advanced melanoma who stopped single-agent anti-PD-1 therapy for objective response or toxicity independent of the line of treatment [27]. The median follow-up after the introduction of treatment was 36.5 months (range 4.6–62.4), and the median follow-up after the discontinuation of treatment was 15.7 months (range 2.5–45.1). Twenty-five (38.4%) patients stopped therapy for CR, 12 (18.5%) for PR/SD and 28 (43.1%) for AEs. The median treatment time was 16.8 months, 21 months, and 7.2 months, for CR, PR/SD, and AE patients, respectively. The median PFS for the whole cohort was not reached. At a median follow-up of 36.5 months from the interruption of treatment, 12 patients (18.5%) relapsed after a median time of 9 months. Of them, 12% had a CR, 16.7% had a PR/SD, and 25% had AEs. The authors found no statistically significant correlations between the disease characteristics and the likelihood of progression, even with the time of treatment, probably due to the small size of the study cohort, as the authors explain. This cohort, with a global recurrence rate of 18.5%, confirmed a long-lasting response after anti-PD-1 cessation regardless of the cause of discontinuation [27]. 

In a recent retrospective, monocenter study, Perez et al. recognized the possibility of discontinuing ICI therapy in patients who have obtained a CR [28]. Of 132 patients, 46 achieved a CR and discontinued treatment after a second lot of radiographic evaluations performed 3 months later. At a median follow-up of 4 years, only 4 (8%) of 46 CR patients experienced disease recurrence, and 100% of patients were alive at the data cut-off. DFS from the end of treatment was 97.5% at 1 year and 94.5% at 30 months. The limitation of this study is that patients were not treated homogeneously. Twenty-three patients were treated with the combination nivolumab plus ipilimumab and 10 patients with the addition of an anti-BRAF or anti-MEK at first progression with ICI therapy [28]. The main features of the studies are summarized in Table 1.

### 3.3. Retreatment

Data regarding re-treatment with anti-PD-1 at recurrence after cessation due to best response or patient/investigator decision are poor and involve very small patient populations.

Already in 2017, Nomura et al. showed that retreatment with nivolumab was an option for selected MM after previous nivolumab treatment. Among eight re-treated patients, two (25%) achieved a PR, and three (37.5%) an SD, with an overall DCR of 62% [29]. In the same year, Blasig et al. reported similar findings in eight MM patients re-treated with anti-PD-1. One of them (12.5%) obtained a PR, while three (37.5%) had an SD (DCR 50%) [30]. In the Keynote 006 study, 13 patients underwent re-treatment with a second course of pembrolizumab. Of them, three obtained a CR, four a PR, three an SD, and one a PD (for two patients, the response assessment was pending), with an overall response rate (ORR) of 54% and a DCR of 77%. Immune-mediated adverse events during the second course were mild to moderate [1]. Jansen et al. [22], in their real-world cohort study of 185 MM patients who electively discontinued anti-PD-1 therapy with pembrolizumab (*n* = 167) or nivolumab (*n* = 18) in the absence of disease progression or TLT, reported relapse in 40 patients (21.6%). Of these, 19 (48%) were re-challenged with pembrolizumab or nivolumab. The BOR at re-treatment was two (11%) CR, four (21%) PR, and five (26%) SD (DCR 58%). Six (32%) patients did not benefit from retreatment, moreover, one patient died before the first response evaluation, and one was not evaluated. Of note, among the nine patients that had a CR with the first course of treatment, four showed an objective response at the re-treatment. The authors concluded that a new course of anti-PD-1 can lead to renewed antitumor activity in a subset of patients [22].

In the real-world, large experience reported by Betof Warner et al. [25], 78 of 396 patients who discontinued treatment for all reasons, including 196 patients for disease progression, were re-treated with single-agent anti-PD-1 therapy (34 patients) or with the combination of ipilimumab plus nivolumab (44 patients). Unfortunately, in this experience the response to re-treatment was infrequent. Only five patients (14.7%) responded to re-treatment with anti–PD-1 therapy including two CR, while eleven (25%) patients responded to re-treatment with the ipilimumab–nivolumab combination, including three CR. No correlation was observed between the response to the initial course of anti–PD1 therapy: only 2 patients of 10 who initially had a CR to the first course of anti-PD-1 responded to re-treatment. The authors came to the conclusion that the long duration of the first-line therapy could induce some resistance mechanisms (e.g., adaptive immune resistance, phenotypic changes of residual tumor cells, antigen processing or presentation, PD-1 expression), allowing the selection of more resistant cellular clones able to resist re-treatment with the same antibodies [25].

More recently, van Zeijl et al. analyzed 27 out of 87 patients (31%) with PD after anti-PD-1 discontinuation who were thus retreated with anti-PD-1 monotherapy. Of them, two (6.7%) had a CR, six (20%) a PR, and nine (30%) an SD. Finally, six (20%) patients had PD and in four (13%) of them, the response status was unknown at the data cut-off [23].

Asher et al. retreated 19 patients with anti-PD-1 at disease relapse. They reported an ORR of 47% and a DCR of 68% including five CR, four PR, and four SD. Two patients progressed and another two had an unconfirmed PD. No correlation was observed between time without treatment and response to re-treatment. All patients were alive at the data cut-off [26].

In the article by Pokorny et al., 13 patients experienced recurrence after first-line anti-PD-1 therapy. Of these, eight were treated with anti-PD-1, including four after loco-regional approaches and one after antiBRAF/antiMEK-targeted therapy. Five of them had an SD and two had a PR (ORR 25% and DCR 87.5%). All but one of the patients were alive at the time of data cutoff [24].

Valentin et al. saw that 12 patients (18.5% of the entire population considered) progressed after a median time of 9 months from checkpoint inhibitors discontinuation [27]. Of them, nine received a second course of treatment. The response to the first course of treatment before discontinuation was CR for three of them and PR/SD for two of them; four discontinued due to AEs. Out of the three patients who discontinued first-course treatment for CR, two had no disease progression (1 CR and 1 SD), and one experienced PD. Of the two patients who stopped for PR/SD, one had no progression and one died of infectious diverticulitis. Of the four patients who discontinued due to AEs, one was in CR after mastectomy without any further therapy, while the other three were still being treated [27].

Of note, when the choice to suspend therapy was due to the occurrence of irAEs, the response rate at the PD-1 was a little higher. Nevertheless, about one-third of patients had a recurrence of the same irAEs at rechallenge with the same ICI. Different irAEs occurred in about 5% of patients. In these cases, rechallenge resulted more frequently for endocrine irAEs and uveitis, and less commonly after pneumonitis [11].

It has been reported that even patients with a history of disease progression in initial checkpoint inhibitor therapy may benefit from rechallenging with checkpoint inhibitors, even if the response is much lower. Reschke et al. reviewed experiences of rechallenging with checkpoint inhibitors in 570 melanoma patients [31], divided into four groups: (1) rechallenge with anti-PD-1 following disease progression in anti-PD-1 therapy; (2) rechallenge with anti-PD-1 and anti-CTLA-4 following disease progression in anti-PD-1 therapy; (3) rechallenge with anti-CLTA-4 following disease progression in anti-CTLA-4 therapy; and (4) rechallenge following toxicity-related treatment discontinuation. In the first group of 85 patients, the mean DCR was 45.8%, and the ORR was 15.5%. The second group of 114 patients showed a mean DCR of 40.6% and an ORR of 20%. In the third group of 182 patients, the mean DCR was 50.9%, with an ORR of 20.4%. Finally, the 189 patients in the fourth group showed a mean DCR of 89.5% and an ORR of 70.2%. Of this last group, 18% of patients showed a recurrence of the same toxicity and 23% reported different adverse events [31].

In conclusion, due to the small number of patients and the short follow-up, the effectiveness of anti-PD-1 re-treatment cannot yet be drawn. Re-treatment had proven to be a reasonable option for selected patients, but further investigations were needed to identify subgroups of patients who can benefit (Table 2).

## 4. Discussion and Conclusions

Answering the question “what is the minimum duration of anti-PD-1 treatment to be carried out before a discontinuation?” is extremely urgent for clinicians, patients, and health economists. Unfortunately, this question is far from being answered due to the absence of controlled prospective clinical trials. In spite of these limits, available data support the hypothesis that most patients who obtain a major response maintain it after treatment cessation [32,33,34].

There are some previous experiences regarding the discontinuation of immunotherapy in melanoma. Specifically, high-dose interleukin-2 and the anti- CTLA-4 agent ipilimumab were associated with complete and durable responses, albeit limited in number, that occurred within only a few months of therapy [35,36]. However, preliminary data from controlled clinical trials do not point in the same direction. The phase-IIIb/IV Checkmate-153 study, specifically designed to compare outcomes in patients with non-small cell lung cancer stopping nivolumab after 12 months vs. continuous therapy, concluded that patients could benefit more from continuous therapy: an exploratory analysis carried out on 252 patients randomly assigned to continuous (*n* = 127) or 1-year fixed-duration (*n* = 125) treatment, at a minimum follow-up of 13.5 months, reported a median PFS of 24.7 months for continuous therapy vs. 9.4 months for 1-year with a median overall survival not reached vs. 32.5 months, respectively [37]. However, these results cannot be translated to melanoma, a disease with different biology and higher responsiveness to immunotherapy. 

Some studies have been designed to specifically answer this question in melanoma. The DANTE (duration of ANti-PD-1 monoclonal antibody treatment in patients with metastatic melanoma) trial is currently ongoing in the UK and is expected to mature in 2027. This is a multicenter phase-III trial that randomizes patients to either discontinue anti-PD-1 therapy at 12 months or continue until disease progression or unacceptable toxicity, or a minimum of 2 years of treatment [38]. Another similar multicenter prospective study, the Safe Stop trial, is ongoing in the Netherlands. The hypothesis is that the early cessation of anti-PD-1 therapy is safe and allows quality-of-life improvement and cost reduction. The early discontinuation of PD-1 blockade upon achieving a CR or PR in patients with MM is compared to patients with CR or PR who completed 24 months of treatment and had an ongoing response at treatment discontinuation [39]. Finally, the ECOG-ACRIN Cancer Research Group designed the PET-STOP trial (NCT04462406), in which the discontinuation is guided by positron emission tomography/computed tomography (PET/CT) imaging and tumor biopsy. This is a phase-II trial investigating how well the biomarkers in PET/CT imaging drive the early discontinuation of anti-PD-1 therapy in patients with stage IIIB-IV melanoma. The experimental group of patients with a negative FDG-PET/CT scan or a positive FDG-PET/CT scan but a negative biopsy for viable tumor discontinue the anti-PD-1 therapy and undergo active surveillance. In the active comparison group, patients with a positive FDG-PET/CT scan and positive biopsy for a viable tumor or a positive FDG-PET/CT scan and biopsy not performed, the patients continue their standard-care anti-PD-1 therapy for 12 months in the absence of disease progression or unacceptable toxicity [40].

While these studies are completed, clinicians need to extrapolate suggestions from currently available data in order to make the right decision for each individual patient. In the registrative phase-III trials, PD-1 blockers were administered for up to two years or longer until unacceptable AEs [1,5,41,42]. Interestingly, durable tumor responses have also been observed after the early discontinuation (<2 years) of PD-1 blockade [1,6,8], not only in patients who achieved a CR [9] but also in patients with a PR or SD [1,10]. 

In some studies, a similar benefit was also reported in the case of response when the discontinuation of treatment was necessary for relevant toxicities [1,9,18,19,43]. Nevertheless, some real-life experiences have reported poorer survival in patients who discontinued due to AEs compared with those who electively discontinued anti-PD-1 therapy [23]. A possible explanation may be that patients who discontinued due to AEs are exposed to a shorter period of therapy. Moreover, most of these patients at the time of discontinuation had not obtained a major response [23]. 

It is emerging that the quality of response has an impact on PFS and OS, both for immunotherapy and targeted therapy [4,13,16,17]. It should also be considered that obtaining a deep and lasting response is often not only the result of the therapy used but also the result of the more favorable characteristics of the patient and of the disease at the start of anti-PD-1 treatment. It is certain that obtaining the best response must be a goal to be pursued with our therapies. For this purpose, locoregional treatments for residual disease or oligoprogression can also be considered in selected patients [44].

In agreement with some studies, which reported a low incidence of relapse after a median follow-up of nearly 2 years from the discontinuation of treatment in patients who received PD-1 blockers for at least 6 months, a maximum 2 years of therapy may be considered sufficient to maintain long term response [1,9,20]. In addition, for patients who have achieved a CR, even a shorter period can be considered without increasing the risk of recurrence, provided that at least 6 months of therapy is completed [22,24]. For patients with PRs with substantial residual tumor burden or patients with SD, treatment for at least 2 years is recommended, although, in some patients, indefinite therapy may be indicated based on the amount of disease evident on imaging and in the absence of relevant toxicity [22,23].

However, it is to be considered that in some northern European countries, such as the Netherlands, the maximum period allowed is 24 months [23]. From the above, it can be inferred that an important parameter influencing the choice to discontinue therapy is the quality of response, in addition to the patient’s willingness and the risk of toxicity.

It is important to consider, however, that many retrospective studies do not use codified criteria for evaluating responses, and that evaluation is often performed on the judgment of the investigator. This is an important bias, especially for multicenter studies in which evaluation is left to the individual investigator.

It should be noted that, despite a major response, about 25–30% of patients are at risk of recurrence in a time period ranging from a few months to some years from treatment cessation, and, when retreated, they respond less than in the first line setting: about 20% of response rate for patients retreated with anti-PD-1 as a single agent and about 30% when used in combination with anti-CTLA-4 [1,21,22,23,24,25,26,27,28,29,30]. It is noteworthy, however, that patients who have CR at discontinuation have a higher probability of response to retreatment (about 50 percent) [1]. The re-challenge after interruption due to side effects seems to induce a slightly greater response of about 30% [11]. Nevertheless, discontinuation experiences are still limited to too-small numbers to draw any firm conclusions.

For these reasons, it is recommended to share the decision to stop therapy with the patient after a deep, balanced discussion with the patient on the risk of relapse, chances of response, and treatment-related toxicities.

The new frontier of immunotherapy is now based on checkpoint inhibitor combinations, with different toxicity profiles than anti-PD-1-only therapy, conditioning more on the choice of time-limited therapy [45].

In conclusion, factors reported to be associated with better off-treatment survival outcomes include CR at treatment discontinuation, patients with CR who have had at least 6 months of therapy, a shorter time from the start of anti-PD-1 treatment to first response, and elective discontinuation vs. discontinuation due to AEs. For now, these parameters can be used to consider anti-PD-1 discontinuation in individual patients. Future prospective trials with precise clinical and biological endpoints are needed to identify the optimal duration of anti-PD-1 therapy. Overall, the quality of response emerges as the most important parameter for decisions about treatment discontinuation. Moreover, most authors suggest not treating patients for more than 2 years, reserving a longer period for carefully selected patients. In fact, some Northern European countries only allow treatment for up to 24 months [23]. Nevertheless, no conclusive data are available for clinicians’ and patients’ choices, most of them being from retrospective studies and regarding heterogeneous experiences in terms of patient populations, time of treatment, response evaluation criteria, and drugs utilized. 

## Figures and Tables

**Table 1:** Type of study and clinical outcomes.

Study	Type of Study	Response Assessment Criteria	Number of Patients Who Discontinued Treatment	BOR, N. Pts (%)	Reason for Discontinuation (N. Pts)	Median Time of Treatment (Months)	Relapse %	Median PFS (Months)	Estimated 24-Month DFS
Keynote 001 [9,20]	phase 1b	RECIST 1.1	91					Not reached	85.8%
				CR, 91 (100)	Joint decision (67) Other ^1^ (24)	23	6%	-	89.9%
						12	-	-	
Keynote 006 [1]	phase 3	RECIST 1.1	103				26%	Not reached	78.4%
				CR, 21 (20.4)	End of protocol	24	24%	Not reached	85.4%
				PR, 69 (67)			23%	Not reached	82.3%
				SD, 13 (12.6)			46%	Not reached	39.9%
Jansen [22]	Retrospective/prospective	irRC	185			12	22%		
				CR, 117 (63)	Joint decision	11	14%	Not reached	-
				PR, 44 (24)		15	32%	Not reached	-
				SD, 16 (9) ^2^		14	50%	16	-
Betof Warner [25]	Retrospective	RECIST 1.1	102	CR, 102 (100)	PD after CR (3)	9.4	22.5%	Not reached	83.3%
					TLT (24)				
					CR (72)				
					Other (3)				
Dimitriou [21]	Retrospective	RECIST 1.1	125	CR, 125 (100)	CR (86)	22	8%	Not reached	-
					Other ^3^ (39)	3	7.7%	Not reached	-
van Zeijl [23]	Retrospective	Physician’s judgment	324	CR ^4^,90 (27.8)	Joint decision (63)	12	17.8%	Not reached	64%
					Other ^5^ (27)				
				PR ^4^,190 (58.6)	Joint decision (98)	13	27.4%	Not reached	53%
					Other ^6^ (92)				
				SD ^4^,44 (13.6)	Joint decision (15)	15	43.2%	10	31%
					Other ^7^ (29)				
Pokorny [24]	Retrospective	RECIST 1.1	52				25%		-
				CR,13 (25)	Joint decision	11.1	15.3%	not reached	
				PR, 28 (53.8)			25%		
				SD, 11 (21.2)			63.3%		
Asher N [26]	Retrospective	Not specified	106			15.2	32%		-
				CR, 80 (75.5)	TLT (60) CR (32) Long-term PR (14)	15.5	23.7%	Not reached	
				PR, 22 (20.7)		12.9	50%	36.5	
				SD, 4 (3.8)		12.4	100%	12.8	
Valentin [27]	Retrospective	RECIST 1.1	65			14.1	18.5%	-	-
					CR (25)	16.8	12%		
					PR/SD (12)	21.2	16.7%		
					TLT (28)	7.2	25%		
Perez [28]	Retrospective	RECIST 1.1	46	CR, 46 (100)	CR (46)	9.6	8%	-	-

CR: complete response; PR: partial response; SD: stable disease; PD: progressive disease; TLT: treatment-limiting toxicity; BOR: best overall response. ^1^ Twelve pts due to AEs, two pts for subsequent PD, seven pts for physician’s decision, three for withdrawal of consent. ^2^ Eight (4%) patients were non-evaluable for BOR. ^3^ Thirty-three patients due to TLT and six due to physician’s decision. ^4^ Response at the time of anti-PD-1 discontinuation. ^5^ Five pts due to patient’s choice, seven due to TLT, other reasons in thirteen pts, unknown in two. ^6^ Seven pts due to patient’s choice, sixty-one due to TLT, other reasons in twenty-two pts, unknown in two. ^7^ Two pts due to patient’s choice, twenty-one due to TLT, other reasons in six pts.

**Table 2:** Response to the second course of immunotherapy in patients who had disease progression.

Study	N. Patients Retreated	ORR, %	DCR, %
Keynote 006 [1]	13	54	77
van Zeijl [23]	27	30	63
Pokorny [24]	8	25	87
Betof Warner [25]	78	21	-
Jansen [22]	19	32	58
Asher [26]	19	47	68
Valentin [27]	9	-	66
Nomura [29]	8	25	62
Blasig [30]	8	12.5	50

## Supplementary Materials

The following are available online at https://www.mdpi.com/article/10.3390/biomedicines10102424/s1, Figure S1: PRISMA 2020 flow diagram for new systematic reviews which included searches of databases, registers and other sources.

## Abbreviations

ICI, Immune checkpoint inhibition; CTLA-4, cytotoxic T lymphocyte antigen-4; PD-1, programmed death 1 receptor; OS, overall survival; PFS, progression-free survival; MM, metastatic melanoma; FDA: Food and Drug Administration; TLT: treatment-limiting toxicity; PD: progression disease; DFS, disease-free survival; SD, stable disease; CR, complete response; PR, partial response; AEs, adverse events; BOR, best overall response; LDH, lactate dehydrogenase; OR, odds ratio; HR, hazard ratio; DCR, disease control rate; ORR, overall response rate.
