# Supplementary material for: Checkpoint Inhibitors Immunotherapy in Metastatic Melanoma: When to Stop Treatment?
Source: Biomedicines. 2022 Sep 28;10(10):2424. doi: 10.3390/biomedicines10102424 (PMC9599026; doi:10.3390/biomedicines10102424)
Supplement: Supplementary file 1 [file biomedicines-10-02424-s001.zip › biomedicines-1795258-supplementary.pdf]

Figure S1: PRISMA 2020 flow diagram for new systematic reviews which included searches of databases, registers and other sources

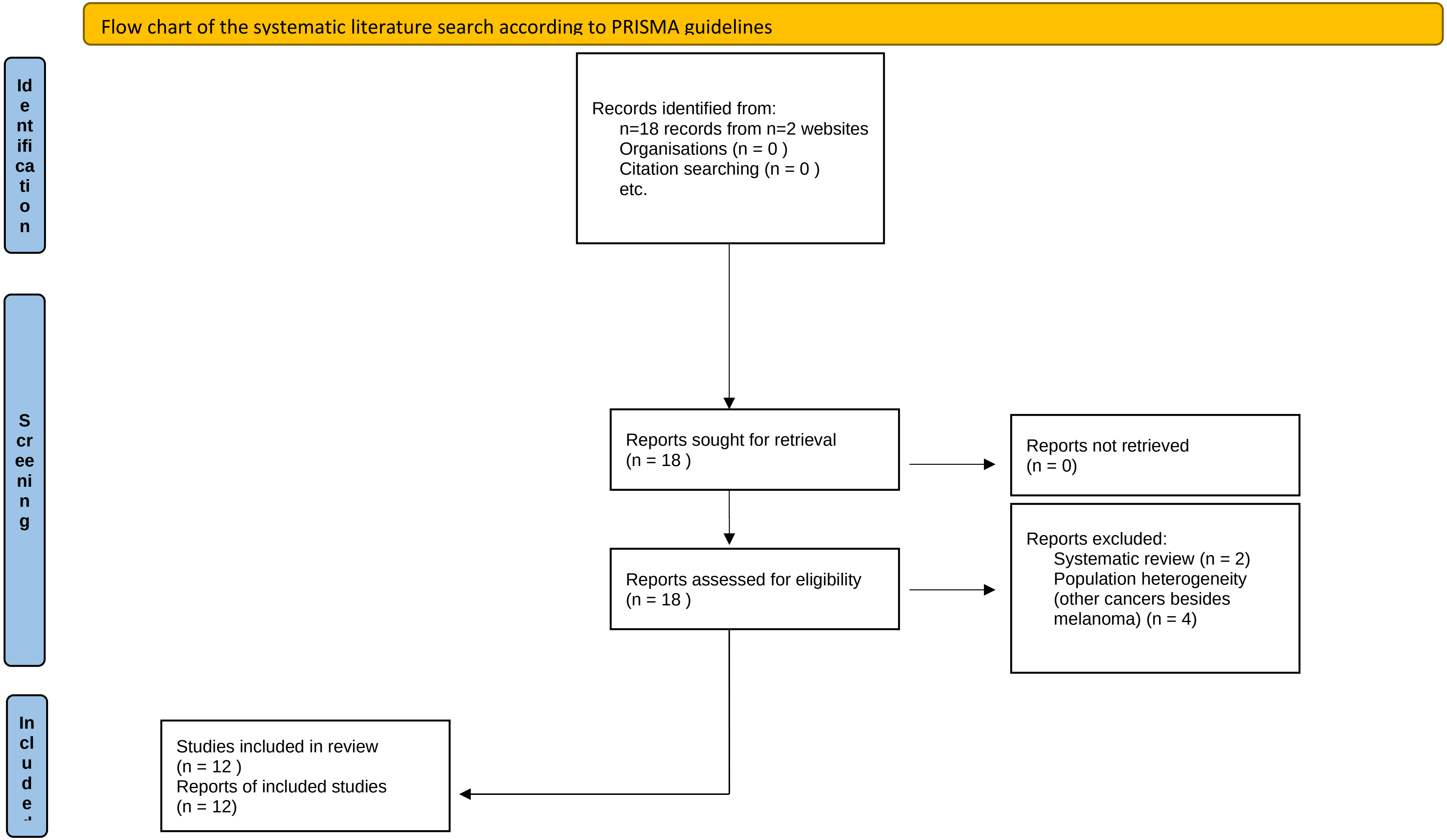

*Consider, if feasible to do so, reporting the number of records identified from each database or register searched (rather than the total number across all databases/registers).
**If automation tools were used, indicate how many records were excluded by a human and how many were excluded by automation tools.

From: Page MJ, McKenzie JE, Bossuyt PM, Boutron I, Hoffmann TC, Mulrow CD, et al. The PRISMA 2020 statement: an updated guideline for reporting systematic reviews. BMJ 2021;372:n71. doi: 10.1136/bmj.n71. For more information, visit: http://www.prisma-statement.org/
